# GPU-Accelerated WPA/WPA2 Credential Recovery as an Enabler for Non-Destructive Drone Neutralization

**DOI:** 10.3390/s26185966

**Published:** 2026-09-21

**Authors:** Yeonjeong Hwang, Junyoung Son

**Affiliations:** School of Computer Science and Engineering, Pusan National University, Busan 46241, Republic of Korea; gmgkgk112@gmail.com

**Keywords:** counter-UAS, WPA/WPA2, GPU-accelerated password cracking, drone security, CUDA, Wi-Fi vulnerability

## Abstract

Drones have become a significant threat in both modern warfare and civilian environments, presenting unprecedented security challenges. While hard kill (physical destruction) defense methods exist to counter threat drones, they remain prohibitively expensive and often result in collateral damage. This study focuses on software-based peaceful neutralization as an anti-drone countermeasure, with particular emphasis on optimizing Wi-Fi credential recovery by leveraging exploitable vulnerabilities in threat drone wireless communications. The methodology included extensive performance evaluation experiments to assess vulnerabilities in drone Wi-Fi networks through GPU-accelerated credential recovery. Across the tested GPU platforms, the proposed implementation achieved an average throughput improvement of 8.78% over the Hashcat baseline, with platform-specific gains of 5.0% on H100, 8.77% on A100, 6.53% on RTX PRO 6000, and 14.8% on RTX 4060. The proposed approach represents a preliminary step toward a cost-effective and damage-minimizing alternative, applicable to Wi-Fi-dependent drone platforms under specific operational conditions. Our experimental results suggest the potential feasibility of credential-recovery-based approaches as an enabling step toward software-based neutralization of Wi-Fi-dependent drone systems. This paper contributes to the emerging field of counter-unmanned aerial systems (C-UAS) by establishing a methodology for credential recovery against Wi-Fi-based drone communications, which may contribute to reducing reliance on physical intervention in applicable scenarios.

## 1. Introduction

An Unmanned Aerial Vehicle (UAV), commonly known as a drone, represents an aircraft that operates without a human pilot aboard, controlled either autonomously or remotely through wireless communication channels. As drone technology has rapidly evolved, these systems have become increasingly sophisticated and interconnected, with their capabilities expanding across diverse fields including military operations, commercial applications, and civilian use. The proliferation of drones in modern warfare has highlighted their potential as strategic assets, particularly due to their precision targeting capabilities, operator protection through distance, and enhanced stealth characteristics compared to conventional weapons systems.

Despite their increasing deployment in sensitive applications, comprehensive security analysis of drone wireless communication protocols remains insufficient [1,2]. This security gap is especially concerning given well-documented vulnerabilities in common wireless protocols such as Wi-Fi. Current encryption implementations are often susceptible to offline cracking attacks [3,4], particularly as modern GPU computational capabilities have dramatically reduced the barriers to sophisticated cryptographic attacks against Pre-shared Keys (PSK). This paper presents a comprehensive security analysis of WPA/WPA2 vulnerabilities in drone wireless communication systems, with a focus on GPU-accelerated credential recovery as an enabler for non-destructive drone neutralization. Our main contributions are as follows:

- We performed an in-depth feasibility analysis of WPA/WPA2 password cracking using modern GPU-accelerated computing environments. Our evaluation leverages state-of-the-art hardware including NVIDIA’s RTX 4060, RTX PRO 6000, A100, and H100 architectures [5] to assess the practical computational requirements for breaking WPA/WPA2 security, demonstrating the dramatically reduced barriers to such attacks and their implications for drone system security.
- We developed and analyzed optimized CUDA implementations for WPA/WPA2 password cracking, achieving a 5% performance improvement over existing state-of-the-art tools. Through systematic evaluation, we identified effective optimization techniques including PRMT-based word generation, vectorization, shared memory utilization [6], and zero-based block removal, while documenting approaches that proved counterproductive.
- We investigated potential post-access neutralization scenarios, examining software-based intervention techniques enabled by successful credential recovery. This analysis provides a comprehensive threat model for Wi-Fi-based drone architectures and informs the development of effective countermeasures.

This research evaluates the practical feasibility of password cracking attacks targeting drone systems, examining how recent advances in GPU architectures have fundamentally altered the security landscape. Our findings highlight critical security gaps in current drone communication systems and provide a foundation for developing more robust security countermeasures. This work serves as an important step toward improving the security posture of commercial drone systems and protecting them against emerging cyber threats.

## 2. Applicability Scope Analysis

Yaacoub et al. [1] classify drone communication into four domains according to the communication target: Drone-to-Drone (D2D), Drone-to-Ground-Station (D2GS), Drone-to-Network (D2N), and Drone-to-Satellite (D2S). Among these, D2GS is based on Wi-Fi/Bluetooth and is largely unencrypted, making it vulnerable to eavesdropping and man-in-the-middle attacks. The proposed framework is applicable to D2GS, but cannot be directly applied to satellite-based D2S links or to non-standardized D2D links.

Furthermore, considering the weight-based UAV classification of Fotouhi et al. [2] (Micro to Large), the proposed framework applies to Micro-to-Small consumer/prosumer drones (e.g., photography and leisure drones) that rely on Wi-Fi-based command-and-control (C2) links, but does not apply to Medium-to-Large military or industrial drones, which typically employ dedicated encrypted RF or satellite links.

When drones are classified according to control mechanism, the proposed framework applies to remotely piloted drones, but is only partially applicable to fully autonomous drones, since the point at which the command signal can be intercepted differs between the two control schemes.

In terms of usage domain, among the civilian, police, military, criminal, and terrorist domains identified in [1], the proposed framework primarily targets the Wi-Fi C2 channel used by commercial and leisure civilian drones, and falls outside the scope of military and law-enforcement domains that rely on cellular (D2N) links or dedicated encrypted links.

From the perspective of attacker capability, among the Low/Medium/High tiers proposed in [2], the framework—which relies solely on commodity cracking tools (e.g., the airodump/aireplay/aircrack toolchain)—covers the Low-to-Medium tiers, and does not extend to the High tier, which requires hardware reverse engineering or side-channel attacks. In addition, the framework presupposes that the attacker is physically located within Wi-Fi range of the drone-GCS link and, unless active de-authentication is incorporated, must wait for a naturally occurring WPA handshake, which constitutes a further practical constraint on its applicability.

Table 1 compares the attack types classified in [1] with the proposed framework to indicate which attacks are directly or indirectly addressed by it. In [2], the risk level of each drone communication type is analyzed, and the proposed framework targets the Wi-Fi link of D2GS, which is rated as high risk.

## 3. Commercial Drone Wi-Fi Security Analysis

Commercial drones increasingly rely on Wi-Fi for control, video transmission, and firmware updates. We surveyed representative consumer and industrial drone platforms to assess their Wi-Fi security configurations, with findings summarized in Table 2.

As shown in the table, the majority of surveyed platforms employ WPA2-PSK as their primary security mechanism, with enterprise models such as the DJI Matrice series adding AES-256 encryption as an additional layer. However, legacy consumer drones—including the Parrot AR.Drone and the default configuration of the Ryze Tech Tello—operate on open networks without any authentication, exposing them to trivial hijacking attacks requiring no specialized hardware.

These findings confirm that WPA2-PSK remains the de facto standard across commercial drone platforms, making the GPU-accelerated cracking methodology presented in this paper directly applicable to a broad range of real-world targets.

## 4. Related Works

In this section, we first analyze the evolution of Wi-Fi security vulnerabilities, from WEP to WPA2, including denial-of-service (DoS) and cracking attacks. We then examine Wi-Fi-based security vulnerabilities in drone communication systems. Also, we will address the evolving status of cracking attack related to advancing GPU specs.

### 4.1. Wi-Fi Network Vulnerability

The evolution of Wi-Fi security protocols represents an ongoing arms race between security mechanisms and emerging attack vectors. The initial Wi-Fi encryption protocol, WEP, exhibited fundamental cryptographic weaknesses that enabled network compromise within 60 s given sufficient packet capture (approximately 85,000 packets), achieving success rates of up to 95% [7]. Its successor, WPA, while designed as an interim solution, inherited vulnerabilities due to its reliance on the Temporal Key Integrity Protocol (TKIP), which proved inadequate against modern attack methods. Also it is revealed that older implementation of WPA is susceptible to side-channel Attacks [8]. Despite employing the more robust Advanced Encryption Standard (AES), WPA2 contains exploitable vulnerabilities in its key management and authentication processes. The 2017 discovery of the Key Reinstallation Attack (KRACK) demonstrated that adversaries could compromise the four-way handshake protocol by manipulating authentication key reinstallation procedures [3]. WPA3 attempted to address these shortcomings through the implementation of Simultaneous Authentication of Equals (SAE), based on the Dragonfly key exchange protocol. However, this introduces two critical challenges for drone operations: first, the computational requirements exceed the capabilities of many resource-constrained drone platforms, and second, the protocol’s handshake mechanism remains vulnerable to denial-of-service attacks. Moreover, both WPA2 and WPA3-transition modes continue to be susceptible to offline pre-shared key (PSK) cracking attacks [9]. While complex passwords—combining special characters, uppercase and lowercase letters, numbers, and exceeding eight characters in length—significantly increase cracking difficulty, advances in GPU technology and GPGPU frameworks have made such attacks feasible within practical timeframes. One of effective DoS attack against Wi-Fi networks is the Deauthentication attack. The deauthentication frame is a legitimate management frame in the 802.11 protocol [10], but it inherently lacks authentication mechanisms. This vulnerability allows attackers to forge these frames and impersonate either an Access Point (AP) or client to forcibly terminate connections. When attackers continuously replay these spoofed frames, the targeted devices repeatedly attempt to reconnect, resulting in a sustained DoS condition. Another significant DoS attack exploits vulnerabilities in the TKIP (Temporal Key Integrity Protocol) algorithm used in WPA. By manipulating packets with specific keystreams, attackers can exhaust computational resources on the receiving node and disrupt network traffic flow. This attack is particularly effective due to TKIP’s implementation of the Michael Message Integrity Check (MIC) and its countermeasures [11].

### 4.2. GPU Advancements and Cracking

**Figure 1:**
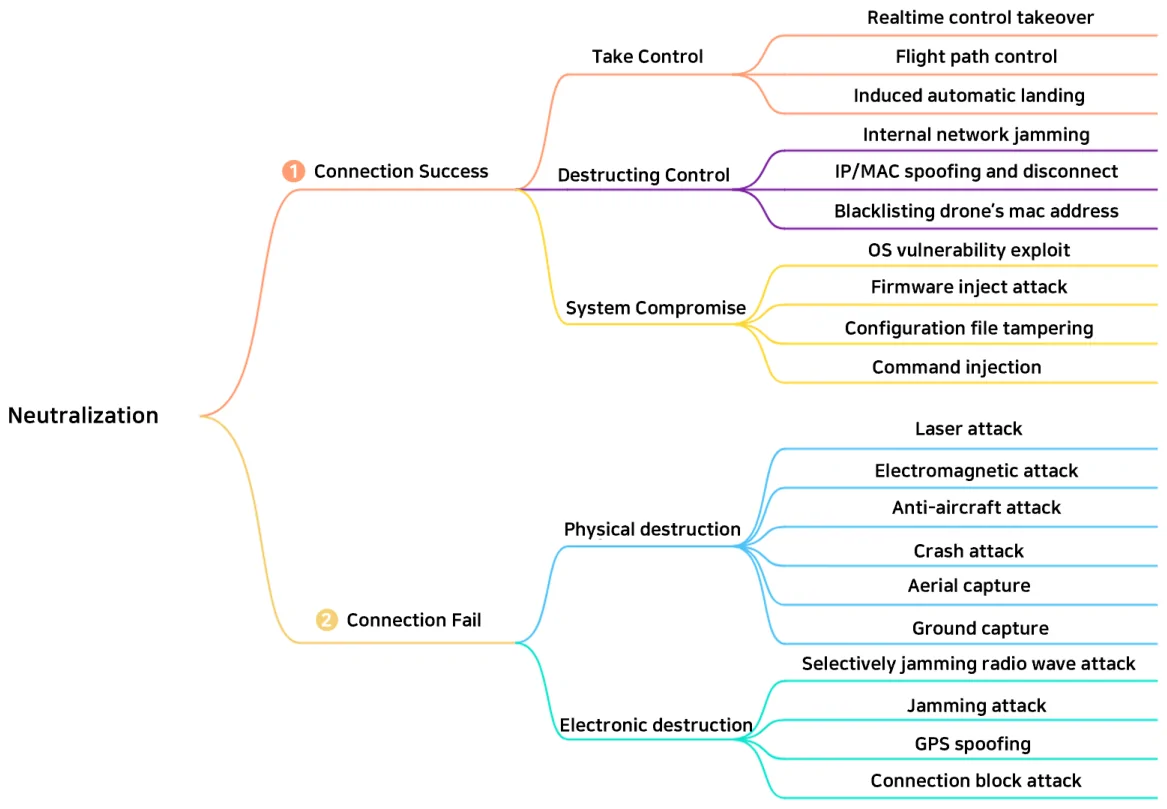
Neutralization Techniques Against Threat Drones.

In ref. [12], the authors analyzed the architectural advantages of GPUs over CPUs for parallel computational tasks such as password cracking. Their study demonstrated that the highly parallel architecture of GPUs, featuring numerous processing cores, enables concurrent execution of identical tasks, resulting in performance improvements of 10 to 100 times compared to CPU-based implementations. Furthermore, ref. [13] emphasized the superior scalability of GPU-based systems, noting that multiple GPUs can be efficiently integrated into a single system to achieve near-linear performance scaling—a capability that remains more constrained in CPU-based architectures. Building on this foundation, ref. [14] developed the first MD5 password cracking tool based on the CUDA GPGPU framework, establishing an early proof of concept for GPU-accelerated cryptographic attacks. In ref. [15], Khader et al. demonstrated the vulnerability of Wi-Fi networks to GPU-accelerated attacks using consumer-grade graphics cards. Their research showed that the NVIDIA RTX 4090 achieved exceptional performance in WPA hash cracking, reaching throughput rates of 19,933 kH/s for PBKDF2-HMAC-SHA1 and 8948 kH/s for PBKDF2-HMAC-SHA256 operations, along with 2720 kH/s for PMKID hash cracking. These figures represented approximately twice the throughput of the second-fastest GPU tested, the AMD RX 7900 XTX, underscoring the growing feasibility of password recovery attacks against Wi-Fi networks using modern consumer hardware.Drone neutralization methods can be broadly categorized based on network accessibility. When an attacker cannot access the drone’s internal network, countermeasures typically rely on physical intervention—such as lasers, EMP devices, or capture nets—or external jamming of GPS, Wi-Fi, and RF control links [16,17]. In contrast, when network access is achievable, for instance by recovering the Wi-Fi PSK, more precise interventions become possible, including control hijacking, MAC blacklisting, and command injection, while minimizing collateral damage [1,18]. Figure 1 illustrates possible neutralization techniques against threat drones, categorized by whether network access to the target drone has been established. The proposed framework specifically addresses the Connection Success branch of this taxonomy, where GPU-accelerated WPA/WPA2 credential recovery serves as the enabling step to achieve network access, subsequently opening pathways to Take Control and other software-based neutralization techniques. The Connection Fail branch, which encompasses physical and electronic destruction methods, is explicitly outside the scope of this work.

## 5. Background

In this section, we present a comprehensive analysis of state-of-the-art GPU architectures optimized for AI/ML workloads, with particular focus on their computational capabilities for cryptographic operations. We provide an in-depth examination of WPA/WPA2 cryptanalysis methodologies, detailing the algorithmic approaches and computational requirements for addressing key performance bottlenecks in the authentication process. Additionally, we explore the CUDA GPGPU programming model and its architectural implications for parallel cryptographic computations. Our analysis encompasses both the theoretical foundation of these attacks and their practical implementation considerations, with specific emphasis on leveraging GPU-accelerated parallel computing for password candidate generation and validation. Furthermore, we evaluate the effectiveness of CUDA’s thread hierarchy and memory management systems in optimizing these cryptographic workflows.

### 5.1. WPA/WPA2 Cracking Process

The Message Integrity Code (MIC) in WPA/WPA2 is derived from the Pre-Shared Key (PSK) through multiple stages of cryptographic operations. Initially, the PSK is processed using PBKDF2-HMAC-SHA1 with the network SSID as a salt value and 4096 iterations to generate a 256-bit Pairwise Master Key (PMK). This PMK serves as the foundation for subsequent key derivation processes. The system then generates a Pairwise Transient Key (PTK) during the 4-way handshake. The PTK derivation utilizes a pseudo-random function PRF-384 with the PMK as its key material. The input to this function concatenates the string “Pairwise key expansion” with ordered MAC addresses of the authenticator and supplicant, along with their respective nonces. The PRF-384 implementation employs HMAC-SHA1 in a three-step process, where each step’s output feeds into the next, ultimately producing a 384-bit key stream. From the generated PTK, the first 128 bits are extracted to form the Key Confirmation Key (KCK), which is used directly in MIC calculations. The actual MIC computation differs between WPA and WPA2: WPA employs HMAC-MD5, while WPA2 uses HMAC-SHA1 truncated to 128 bits. In both cases, the KCK serves as the key for the HMAC operation, with the input being the EAPOL frame (having its MIC field set to zero). The resultant MIC provides cryptographic assurance of message integrity throughout the authentication process. The verification of Wi-Fi Protected Access (WPA/WPA2) credentials can be accomplished through a password recovery attack that leverages captured handshake packets. This attack methodology exploits the deterministic nature of the MIC calculation process, allowing attackers to validate password candidates without active network interaction. When an attacker obtains the necessary parameters from captured handshake packets—specifically the SNonce, ANonce, and network SSID—along with the corresponding MIC value, they can execute an offline dictionary attack by systematically generating candidate MICs from potential passwords. The attack procedure involves reconstructing the entire key derivation chain for each password candidate, starting with the computationally intensive PBKDF2 function that involves 4096 iterations of HMAC-SHA1 operations. This process generates the PMK, which is then used in subsequent PRF-384 calculations to derive the PTK and ultimately compute the MIC. The computational overhead of this attack is primarily attributed to the cryptographic primitives employed in the protocol, particularly the repeated application of SHA1 and HMAC-SHA1 within the PBKDF2 function. The intentional computational complexity introduced by these 4096 iterations serves as a defensive mechanism against high-speed password recovery attempts, making it the principal performance bottleneck in the credential recovery process. This computational barrier, while designed as a security feature, remains a crucial point of consideration in analyzing the protocol’s resistance to offline dictionary attacks. The effectiveness of this protection mechanism is directly proportional to the computational resources required for each password attempt, fundamentally affecting the feasibility and time requirements of a successful attack. To summarize, Cracking process of WPA/WPA2 is mainly based on complexity of SHA1 consists of bit rotation, XOR, addition, and assignment operations. These operations are optimized by most modern compilers, so they do not significantly impact the overall calculation speed of the SHA1 hash function.

### 5.2. CUDA GPGPU

CUDA (Compute Unified Device Architecture) represents a specialized parallel computing platform and programming model designed specifically for NVIDIA Graphics Processing Units (GPUs). While CUDA’s hardware-specific nature restricts its compilation to environments with the CUDA toolkit installed, this specialization enables optimal utilization of NVIDIA GPU architectures through direct access to GPU-specific features including cores and thread management capabilities.

In contrast to OpenCL (Open Computing Language), which prioritizes broad hardware compatibility across diverse computing platforms, CUDA’s focused development for NVIDIA hardware facilitates more comprehensive exploitation of state-of-the-art GPU architectures. This hardware-specific optimization allows developers to leverage advanced features and architectural improvements in contemporary NVIDIA GPUs more effectively. The trade-off between CUDA’s hardware-specific optimization and OpenCL’s platform independence illustrates a fundamental consideration in parallel computing: the balance between performance optimization and cross-platform compatibility.

The specialized nature of CUDA provides developers with deeper access to the underlying hardware architecture, enabling more fine-grained control over GPU resources and potentially superior performance optimization for NVIDIA platforms, despite its more restricted deployment environment compared to OpenCL’s universal approach.

### 5.3. GPU Architecture Comparison

Modern GPU architectures span from data center accelerators to consumer graphics cards, each optimized for different workloads. This section compares four representative GPUs: NVIDIA’s A100 and H100, designed for high-performance computing and AI training; the RTX 4060, a consumer-grade GPU for general-purpose graphics; and the RTX PRO 6000, a professional GPU optimized for data-center-scale AI and visualization workloads.

NVIDIA’s A100 Tensor Core GPU, built on the Ampere GA100 architecture, features 6912 CUDA cores and 432 third-generation Tensor Cores across 108 Streaming Multiprocessors (SMs), fabricated using TSMC’s 7 nm N7 process. The GPU incorporates 40 GB or 80 GB of HBM2e memory with a 5120-bit interface, providing up to 2039 GB/s bandwidth. It achieves up to 156 TFLOPS in FP64 and 312 TFLOPS in FP32 operations, with structured sparsity support allowing 2:1 sparse matrix processing. The A100 introduces Multi-Instance GPU (MIG) technology, enabling partitioning into up to seven isolated GPU instances with QoS guarantees. The architecture features third-generation NVLink technology, delivering 600 GB/s bidirectional bandwidth across twelve connections.

The H100 GPU, built on the Hopper GH100 architecture, features 16,896 CUDA cores and 528 fourth-generation Tensor Cores across 132 SMs, using TSMC’s 4N process. The GPU employs 80 GB of HBM3 memory with a 5120-bit interface, delivering 3.35 TB/s bandwidth and operates at 700 W TDP. It provides 256 KB per SM and a 50 MB L2 cache. The GPU achieves 67 TFLOPS in FP64, 134 TFLOPS in FP32, and up to 989 TFLOPS in FP8 operations with its Transformer Engine, supporting 2:4 structured sparsity patterns. Enhanced MIG technology enables up to seven isolated GPU instances, while fourth-generation NVLink provides 900 GB/s bidirectional bandwidth with PCIe Gen5 support.

The RTX PRO 6000 Blackwell Server Edition, built on the Blackwell GB202 architecture, features 24,064 CUDA cores and 752 fifth-generation Tensor Cores across 188 SMs, also using TSMC’s 4N process. The GPU employs 96 GB of GDDR7 memory with a 512-bit interface, delivering approximately 1597 GB/s bandwidth. Operating at approximately 600 W TDP, it supports PCIe Gen5 connectivity and incorporates DLSS 4 Multi Frame Generation technology, providing enhanced AI-accelerated computing capabilities for high-performance data center and professional visualization applications.

The RTX 4060, based on the Ada Lovelace AD107 architecture, features 3072 CUDA cores and 96 fourth-generation Tensor Cores across 24 SMs, manufactured on TSMC’s 4N process. The GPU incorporates 8 GB of GDDR6 memory with a 128-bit interface, providing 272 GB/s bandwidth. With a 115 W TDP and 24 MB L2 cache, the RTX 4060 supports PCIe Gen4 connectivity and includes DLSS 3 technology for AI-accelerated graphics rendering, targeting mainstream gaming and content creation workloads.

Table 3 provides a comprehensive comparison of these GPU architectures. The data center-class GPUs (A100 and H100) feature high-bandwidth HBM memory and NVLink interconnects, while all three professional/data-center GPUs (A100, H100, and RTX PRO 6000) support Multi-Instance GPU (MIG) technology for multi-tenant workloads, making them suitable for large-scale AI training and HPC applications. Notably, RTX PRO 6000 diverges from this pattern by relying on GDDR7 memory and PCIe-only interconnects rather than HBM and NVLink, reflecting its hybrid design that bridges professional visualization and data-center AI workloads. In contrast, the consumer-grade RTX 4060 utilizes GDDR6 memory with substantially lower power consumption and incorporates DLSS technology for graphics-intensive applications. The progression from A100 to H100 demonstrates significant improvements in memory bandwidth (64% increase) and AI performance through specialized Transformer Engine capabilities.

## 6. Experimental Setup

**Figure 2:**
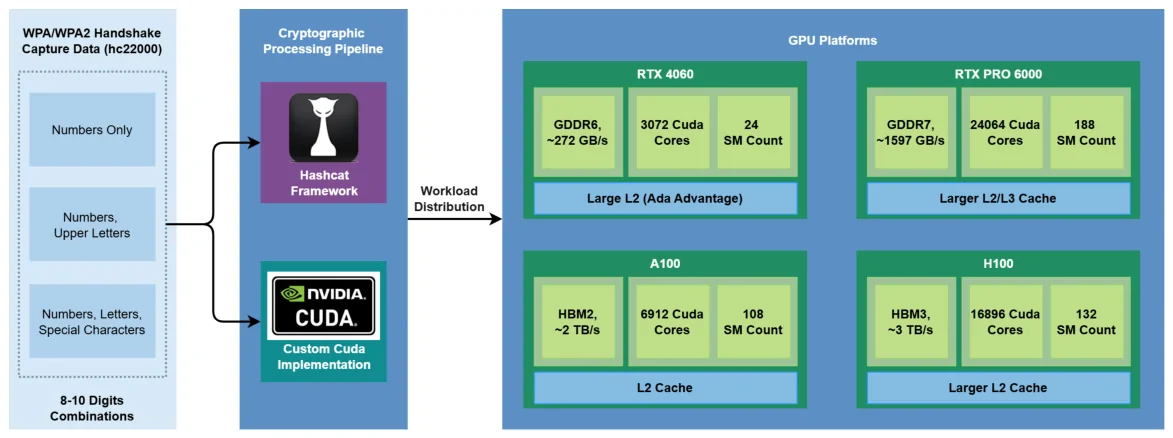
Overall Enviroment.

We developed a CUDA-based WPA/WPA2 password recovery tool to evaluate the feasibility of credential extraction in contemporary GPU environments. Our implementation incorporates several key optimization strategies paralleling those employed in hashcat. The first significant optimization involves complete loop unrolling of the cryptographic primitives: SHA1, HMAC-SHA1, and PBKDF2-HMAC-SHA1. This transformation from iterative to fully unrolled implementations yielded substantial performance improvements, demonstrating approximately 2.8 times faster execution compared to the loop-based implementations. The second major optimization focuses on detailed implementations of critical SHA1 computation components. We meticulously recreated hashcat’s implementation style for the ROTL32 function and SHA1 round operations. Additionally, we developed an inline PTX assembly implementation of the SHA1 word generation function, which demonstrated a 5% performance improvement compared to conventional word generation techniques. On our CUDA implementation, we applied architecture-specific optimization techniques. While Hashcat demonstrates good compatibility to any CPU or GPU platforms, its OpenCL implementation limits access to hardware-specific optimizations. Although OpenCL provides broader compatibility across different computing platforms, it cannot fully utilize the latest NVIDIA GPU features, such as the SM Clustering capability in the Hopper architecture. Therefore, our primary objective was to investigate and implement detailed, architecture-specific optimizations tailored for each state-of-the-art GPU environment. Our experimental methodology can be summarized as follows. We conducted our performance evaluation by implementing both our CUDA code and the Hashcat source code across FOUR distinct server environments, each equipped with a different NVIDIA GPU architecture: Hopper(H100), Ampere(A100), Blackwell(RTX PRO 6000), and Ada Lovelace(RTX 4060). H100, A100, and RTX PRO 6000 represent the state-of-the-art computing hardware widely deployed in modern data centers, particularly for AI/ML workloads and high-performance computing applications. In contrast, the RTX 4060 is a consumer-grade GPU primarily targeted at gaming and general-purpose personal computing. Overall environmental setting can be seen in Figure 2. A summary of the experimental environment is presented in Table 4.

### 6.1. H100 Clustering Architecture

NVIDIA’s Hopper architecture introduces SM (Streaming Multiprocessor) Clustering which can be significant advancement in GPU parallel processing design. This feature enables multiple SMs to operate as unified clusters with shared resources and high-speed communication channels. The clustering mechanism implements shared L1 cache among grouped SMs and dedicated inter-SM communication paths, substantially reducing data movement overhead and latency. This specific architecture of H100 particularly benefits workloads with intensive data sharing requirements where multiple thread blocks need to exchange data frequently. In case of WPA/WPA cracking, the updated pbkdf2 state need to be shared between threads, thus clustering can be beneficial in terms of memory access of iterative pbkdf2 process. We implemented clustered pbkdf2 calculation, where only thread with highest rank in cluster (leader thread of cluster) initialize and create data that needs to be shared between threads.

#### 6.1.1. WPA Handshake Collection

As part of the attack process, WPA handshake packets were obtained by performing deauthentication attacks against WPA/WPA2-enabled access points. The captured handshake packets contained Message Integrity Codes (MICs) generated from various Pre-Shared Key (PSK) combinations, including numeric-only passwords and mixed combinations of numbers, alphabet letters, and special characters. Our analysis focused on PSKs ranging from 8 to 10 characters in length, as 8 characters represent the minimum WPA password requirement, while longer passwords exponentially increase the search space and computational time required for successful cracking attempts. The PSK patterns and their corresponding search spaces are presented in Table 5.

#### 6.1.2. CUDA-Based WPA/WPA2 Password Recovery Tool

To evaluate the feasibility of credential extraction in contemporary GPU environments, a CUDA-based WPA/WPA2 password recovery tool was implemented. Performance was evaluated using two metrics: throughput, defined as the number of password hash attempts processed per minute (H/m), and completion time, defined as the total elapsed time required to exhaust the entire PSK search space for a given character set and length combination. The performance comparison was conducted against Hashcat (v6.2.6) under identical experimental conditions, using the same WPA/WPA2 handshake capture file, character set configurations, and GPU hardware. Our implementation demonstrates an average 8.78% performance improvement over Hashcat across all tested GPU platforms, with platform-specific gains detailed in Section 7. This superior performance was achieved through optimization of the CUDA implementation, including vectorization techniques utilizing CUDA’s built-in vectorized data types (uint4 for 4 combined 32-bit unsigned integers). Our implementation incorporates several optimization strategies paralleling those employed in hashcat. The first significant optimization involves complete loop unrolling of the cryptographic primitives: SHA1, HMAC-SHA1, and PBKDF2-HMAC-SHA1. This transformation from iterative to fully unrolled implementations yielded substantial performance improvements, demonstrating approximately 2.8 times faster execution compared to the loop-based implementations. The second major optimization focuses on detailed implementations of critical SHA1 computation components. The ROTL32 function and SHA1 round operations were implemented following the style of hashcat.

#### 6.1.3. PRMT-Based Word Generation

The PRMT (permute) instruction in NVIDIA GPUs demonstrates superior performance over traditional bitwise operations for byte reordering. While conventional methods require multiple operations (four shifts and three OR operations) to construct a 32-bit word, PRMT accomplishes the same transformation with a single hardware-accelerated instruction. When configured with mask 0x0123 for big-endian conversion, PRMT reduces instruction overhead by approximately 85%. Additionally, the PRMT implementation’s use of 32-bit aligned data (block32) enables more efficient memory access patterns compared to byte-wise operations, leading to better utilization of GPU memory bandwidth. This combination of reduced instruction count and optimized memory access patterns results in significantly improved performance for byte reordering operations in cryptographic implementations.

#### 6.1.4. Zero-Based Block Removal

Optimization of the HMAC computation process was achieved by eliminating redundant XOR operations in the second blocks. As documented in prior research [19], the second block (u2) of HMAC is dependent on the SHA-1 hash result from the previous block computation, which introduces a substantial zero-filled region in the SHA-1 word blocks. As mentioned in [19], all substitution and XOR operations involving these zeroed blocks were computationally unnecessary. (6–14th Word Block) By systematically removing these superfluous operations, additional performance gains were achieved. This targeted elimination of redundant operations represents an important structural improvement to the algorithm’s execution efficiency.

#### 6.1.5. Shared Memory

Shared memory is a high-speed memory resource accessible to all threads within the same block. This on-chip memory allows threads to access data at speeds comparable to L1 cache, which is significantly faster than global memory access. Our performance analysis of the PBKDF2 implementation revealed substantial improvements when allocating frequently accessed data to shared memory. Specifically, placing the ESSID and message buffers for HMAC-SHA1 operations in shared memory yielded performance gains. Since these data structures are repeatedly accessed throughout the PBKDF2 iteration loop, moving them to shared memory reduced memory latency and improved overall computational efficiency.

#### 6.1.6. Other Optimization Techniques

Another optimization technique investigated was the partial unrolling of the PBKDF2 function. While complete unrolling of the SHA1 and HMAC-SHA1 functions is feasible, unrolling the entire 4096-iteration loop in PBKDF2 would introduce significant overhead during both compilation and runtime. A partial unrolling strategy was implemented using CUDA’s pragma unroll directive for the nested loops. Contrary to our expectations, this approach resulted in decreased performance, with approximately 15% slower execution time compared to the non-unrolled version, even when limiting unrolling to just two iterations.

This performance degradation can be attributed to three main factors. First, the increased register pressure significantly impacted performance. The unrolling caused each thread to require more registers, exceeding the GPU’s register limit and forcing local memory spilling into slower global memory. Second, the instruction cache efficiency decreased as the unrolled code size expanded, leading to reduced instruction cache hit rates, particularly affecting complex functions like HMAC-SHA1. Third, the unrolled implementation introduced potential thread divergence within warps, as the expanded code contained more branching points, causing threads within the same warp to potentially follow different execution paths.

Additionally, specific rotation function implementations for SHA1 showed lower performance when compiled with nvcc. While SHA1 only uses 1, 5, and 30-bit rotations, implementing these using CUDA’s __funnelshift library function decreased the SHA1 calculation speed. This suggests that the compiler can best optimize the original bitwise rotation operations.

The vectorization of SHA1 transition functions using internal types of Cuda(uint4), and the implementation of endian transitions using Parallel Reduction and Mapping Techniques (PRMT) resulted in a modest 1% performance improvement. Further refinements to the verification process, specifically in the comparison between the original Message Integrity Code (MIC) and the computed MIC values, provided additional enhancements. By allocating result bytes with cudaHostAlloc and implementing asynchronous device-to-host memory transfers via cudaMemcpyAsync, an additional performance gain was achieved.

## 7. Results & Comparison

**Figure 3:**
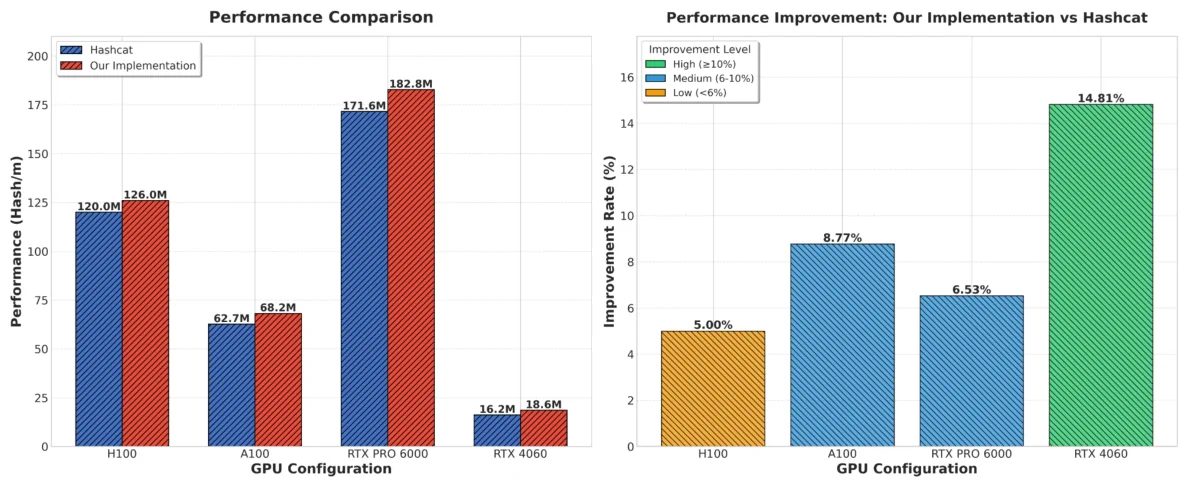
Throughput Comparison Across GPU Platforms.

**Figure 4:**
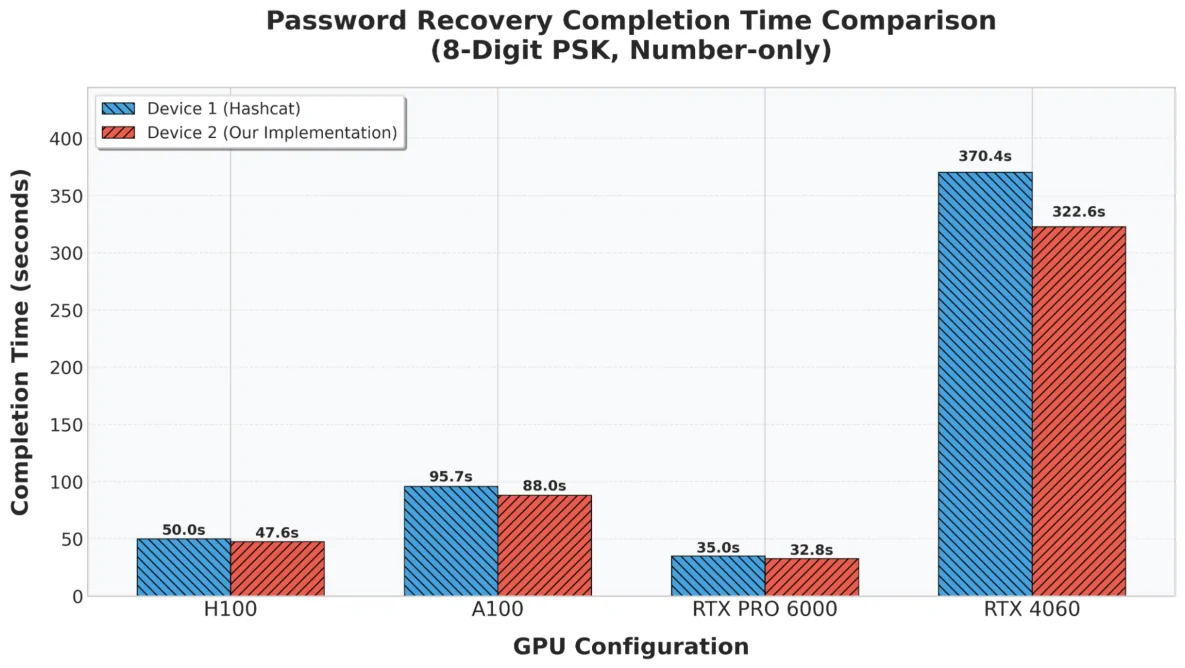
Completion Time Comparison Across GPU Platforms.

A comparison with Hashcat was conducted to evaluate the throughput of the implementation. Among all tested configurations, the H100 GPU demonstrated superior performance, with our code achieving approximately 200,000,000 hashes per second on a single H100 GPU. Figure 3 illustrates comparative throughput measurements across different GPU configurations, including setup of H100, A100, RTX PRO 6000, RTX 4060 architectures. Figure 4 illustrates the completion time comparison across various GPU platforms.

The estimated completion times using 4 × H100 GPUs for various PSK combinations are presented in Table 6. The three character set combinations were selected to represent increasing levels of password complexity, following common password policy recommendations [20]. Numeric-only passwords represent the minimum complexity case, while the combination of numbers and uppercase letters reflects intermediate complexity. The inclusion of all letters and symbols represents the maximum complexity recommended by standard security policies for WPA2 PSK configuration. These results emphasize that password complexity remains a crucial defense against brute-force attacks, and the currently recommended minimum length of 8 characters proves insufficient for security. Our findings demonstrate that passwords limited to a single character set (e.g., numerics only) can be compromised within a feasible timeframe at the minimum length. Only passwords utilizing a combination of numbers, uppercase and lowercase letters, and special characters provide adequate protection against modern GPU-accelerated attacks. From a non-destructive neutralization perspective, these results indicate that threat drones employing weak PSK configurations are susceptible to credential recovery within operationally relevant timeframes. Successful credential recovery enables network access to the target drone, thereby facilitating software-based neutralization through the Connection Success pathways illustrated in Figure 1, without requiring any physical intervention.

Fixed-wing drones can achieve an average flight speed of 80 km/h, while rotary-wing drones typically operate at 60 km/h. In South Korea, sensitive facilities are protected by 18 km flight restriction zones. When attacks originate from outside these zones, the total time required to reach the facilities is approximately 13 min 30 s for fixed-wing drones and 18 min for rotary-wing drones. The tool developed in this paper demonstrates significant performance improvements across different GPU architectures. On the H100 GPU, our implementation achieves a 5% increase in processing throughput (from 120 M to 126 M hash/m), resulting in a 4.76% reduction in cracking completion time compared to conventional cracking tools (hashcat). More notably, the RTX 4060 shows a remarkable 14.81% increase (from 16.2 M to 18.6 M hash/m). On average, our implementation achieves an 8.78% improvement across all tested GPU configurations. The performance comparison was conducted by running both implementations against the same WPA/WPA2 handshake capture, using identical wordlist configurations and character set combinations (Numeric, Numeric + Upper Letters, Numeric + All Letters + Symbols) across 8–10-digit PSK lengths. Throughput was measured in hashes per minute (H/m) and completion time was recorded for each configuration on the same GPU hardware under identical system conditions. Hashcat version 6.2.6 was used as the baseline for comparison.

Consequently, when considering automated neutralization systems, our approach can process an additional 6 M password hash attempts per minute on an H100 GPU, and up to 4 M additional attempts per minute on an RTX 4060. This enhanced processing capability enables credential recovery of shorter and simpler PSK configurations within operationally relevant timeframes, thereby expanding the scope of software-based neutralization opportunities. Completion time encompasses not only the duration required to successfully recover a password, but also the time invested in generating rainbow hash tables for random PSKs. Once a rainbow table has been obtained, running through all hash lists requires minimal processing time. In the experimental evaluation, it was demonstrated that for an 8-digit PSK combination search space, all possible PSKs could be tested within approximately 10 s on high-end GPUs like the H100, and within 38 s on consumer-grade GPUs like the RTX 4060. These findings suggest that hash cracking attacks against 8–9-digit PSKs are feasible with current technology across various GPU price points. Furthermore, the continuous advancement of GPU processing capabilities, as evidenced by the superior performance of the latest RTX 4060, indicates that these vulnerabilities will become increasingly exploitable in the future.

## 8. Limitations

While the proposed framework demonstrates promising results for GPU-accelerated WPA/WPA2 credential recovery, several limitations must be acknowledged.

Applicability scope. The applicability of the proposed framework is inherently limited to a subset of commercial drone platforms, as discussed in detail in Section 2. In particular, drones employing proprietary RF protocols, satellite links, or dedicated encrypted channels remain outside the scope of this work.

Physical and operational constraints. Successful execution of the framework requires the attacker to be physically located within Wi-Fi range (typically ∼100 m) of the target drone’s ground control station link. Furthermore, unless active de-authentication is incorporated, the attacker must wait for a naturally occurring WPA handshake, which may not be feasible in time-critical scenarios.

Password complexity. As shown in Table 6, passwords combining numbers, uppercase and lowercase letters, and special characters of 8 or more characters remain computationally infeasible to crack within operationally relevant timeframes, even with high-end GPU hardware. Additionally, the estimated completion times are based on uniform search space enumeration and do not reflect real-world password distributions, which may include commonly used patterns that could reduce or extend actual cracking times.

Experimental scope. The performance evaluation was conducted in a controlled laboratory environment using a single host system. End-to-end validation—encompassing handshake capture from an active drone, credential recovery, network access, and subsequent neutralization—was not performed in this study and remains a subject for future work.

Ethical and legal considerations. All experiments were conducted in a controlled environment using access points and handshake captures obtained with explicit authorization. The techniques described in this paper are intended solely for defensive research and authorized counter-UAS operations. Unauthorized interception or cracking of Wi-Fi credentials is illegal under applicable laws and regulations.

## 9. Conclusions

This paper presented an analysis of WPA/WPA2 vulnerabilities in commercial drone systems and demonstrated performance improvements in GPU-accelerated credential recovery, representing a potential enabling step toward non-destructive drone neutralization in applicable scenarios. Through analysis of over 150 commercial drones, we identified widespread reliance on WPA/WPA2-protected Wi-Fi networks as a primary communication channel, confirming the applicability of the proposed framework to a significant subset of commercial drone platforms.

The CUDA-based password recovery tool achieved measurable performance improvements over Hashcat (v6.2.6): 5% on H100 GPUs, 8.77% on A100, 6.53% on RTX PRO 6000, and 14.8% on RTX 4060, with an average 8.78% enhancement across all tested platforms, through optimization techniques including PRMT-based word generation, complete loop unrolling, zero-based block removal, and strategic shared memory utilization.

Our experimental results demonstrate that threat drones employing weak PSK configurations are susceptible to credential recovery within operationally relevant timeframes. Notably, 8-digit numeric passwords can be recovered in approximately 11 s on H100 GPUs, suggesting that network access may be achievable within the 13.5–18 min flight time of drones approaching restricted zones, potentially enabling subsequent software-based neutralization under favorable conditions. Furthermore, the effectiveness of consumer-grade GPUs suggests that credential recovery performance is not strictly limited to entities with access to expensive data center hardware. While this study focused on WPA/WPA2 credential recovery, the methodology established here provides a foundation for future research into counter-UAS systems. Future work should expand to include post-compromise neutralization scenarios, investigation of WPA3 in resource-constrained drone platforms, and exploration of non-Wi-Fi drone communication protocols. This research contributes to the emerging field of C-UAS by providing empirical evidence of exploitable vulnerabilities in commercial drone Wi-Fi communications across diverse GPU platforms.

The widespread reliance on WPA2-PSK across commercial drone platforms, as identified in Table 2, highlights the urgent need for stronger security practices. Drone manufacturers should consider enforcing minimum PSK complexity requirements (e.g., 10+ characters with mixed character sets) and transitioning to WPA3 where hardware capabilities permit. Network-level countermeasures such as VPN tunneling and MAC filtering should also be considered as complementary defenses.

## Figures and Tables

**Table 1:** Gap analysis: applicability of the proposed WPA offline-cracking framework against the attack taxonomy of Yaacoub et al. [1].

#	Attack Type	Nature	Existing Countermeasures	Relevance to Framework	Scope
1	Malware Infection	Infection	Hybrid lightweight IDS; access control, system integrity, MFA	Possible only as a follow-on stage after intrusion	Indirect
2	Backdoor Access	Infection	Hybrid lightweight IDS, vulnerability assessment; robust MFA	May be installed after successful penetration	Indirect
3	Social Engineering	Exploitation	Awareness raising, operator training	No human factor; purely technical cracking	N/A
4	Baiting	Exploitation	Awareness raising, operator training	No human factor involved	N/A
5	Injection/Modification	Exploitation	ML-based IDS, timestamping; message auth., digital signatures	Only as a post-intrusion tampering stage	Indirect
6	Fabrication	Exploitation	Privilege assignment; MFA, digital signatures	Only as a post-intrusion false-data stage	Indirect
7	Reconnaissance	Info. gathering	Hybrid lightweight IDS; encrypted traffic	Not part of the capture-and-crack workflow	N/A
8	Scanning	Info. gathering	Lightweight IDS, honeypots; encrypted traffic	Pre-intrusion technique, distinct from framework	N/A
9	Three-Way Handshake	Interception	Traffic filtering, blocking unused ports	Analogous to WPA 4-way handshake capture	**Core**
10	Eavesdropping	Interception	Communication/traffic security, secure links	Corresponds to packet-sniffing/capture stage	**Core**
11	Traffic Analysis	Interception	Communication/traffic security, secure links	Overlaps with analysis of captured traffic	Indirect
12	Man-in-the-Middle	Auth.	Lightweight hybrid IDS; MFA + lightweight crypto-auth.	Enabled once attacker gains network access	Indirect
13	Password Breaking	Cracking	Lightweight IDS; strong, periodic passwords, strong encryption	Cracking target itself (WPA passphrase)	**Core**
14	Wi-Fi Aircrack	Cracking	Physical-layer lightweight IDS; strong, periodic passwords	Identical to airodump/aireplay/aircrack chain	**Core**
15	Wi-Fi Jamming	Jamming	Frequency hopping, band change	No jamming involved (passive capture only)	N/A
16	De-Authentication	Jamming	Frequency hopping, band change	Active deauth not included; a limitation	Optional
17	Replay	Jamming	Frequency hopping, timestamping	Retransmission is a separate technique	N/A
18	Buffer Overflow	Jamming	Frequency hopping, band change	Unrelated attack mechanism	N/A
19	Denial of Service	Jamming	Frequency hopping, band change	Availability attack, not confidentiality-bypass	N/A
20	ARP Cache Poison	Jamming	Frequency hopping, band change	Only as post-intrusion lateral movement	Indirect
21	Ping-of-Death	Jamming	Frequency band change	Unrelated attack mechanism	N/A
22	GPS Spoofing	Jamming	Return-to-base, band change	Targets GPS link, not the Wi-Fi control link	N/A

**Legend:** 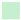 Core, 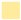 Indirect/follow-on, 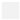 Not applicable.

**Table 2:** Wi-Fi Security Protocols of Representative Commercial Drones.

Model	Transmission	Wi-Fi Usage	Security
DJI Mavic 3 (China)	OcuSync 3.0+	File Transfer, Firmware	WPA2
DJI Air 2S/Mini 3 Pro (China)	OcuSync 3.0	File Transfer, Firmware	WPA2
DJI Mavic Air 1/Mini SE (China)	Enhanced Wi-Fi	Control, Video	WPA2
DJI Spark (China)	Wi-Fi	Control, Video	WPA2
DJI Mavic 3E/Matrice (China)	O3 Enterprise	Local Data, Firmware	WPA2+AES-256
Parrot Anafi USA (France)	Wi-Fi	Control, Video	WPA2
Parrot Bebop (France)	Wi-Fi	Control, Video	WPA2
Parrot AR.Drone (France)	Wi-Fi	Control, Video	Open
Skydio 2/2+ (USA)	Wi-Fi	Control, Video	WPA2-PSK
Skydio X2/X10 (USA)	Wi-Fi/Skydio Link	Network, Dock Ops	WPA2-PSK
Yuneec Typhoon/H520 (China)	Wi-Fi	Control, Video	WPA2-PSK
Autel EVO Series (China)	Wi-Fi/SkyLink	Internet, Data	WPA2
Ryze Tech Tello (China)	Wi-Fi	Full Control	Open ^†^

^†^ Default configuration; WPA2 configurable.

**Table 3:** Comparison of GPU Architectures.

Specification	H100	A100	RTX PRO 6000	RTX 4060
Architecture	Hopper (GH100)	Ampere (GA100)	Blackwell (GB202)	Ada Lovelace (AD107)
Process Node	TSMC 4N	TSMC 7nm (N7)	TSMC 4N	TSMC 4N
CUDA Cores	16,896	6912	24,064	3072
Tensor Cores	528 (4th gen)	432 (3rd gen)	752 (5th gen)	96 (4th gen)
Streaming Multiprocessors	132	108	188	24
Memory Type	HBM3	HBM2e	GDDR7	GDDR6
Memory Capacity	80 GB	40/80 GB	96 GB	8 GB
Memory Interface	5120-bit	5120-bit	512-bit	128-bit
Memory Bandwidth	3350 GB/s	2039 GB/s	1597 GB/s	272 GB/s
L2 Cache	50 MB	40 MB	128 MB	24 MB
FP64 Performance	67 TFLOPS	9.7 TFLOPS	-	-
FP32 Performance	67 TFLOPS	19.5 TFLOPS	120 TFLOPS	15.11 TFLOPS
FP8 Performance	3958 TFLOPS	-	2 PFLOPS	60.46 TFLOPS
Interconnect	NVLink 4.0	NVLink 3.0	PCIe Gen5	PCIe Gen4
Bandwidth (Interconnect)	900 GB/s	600 GB/s	-	-
Special Features	MIG (7 instances),	MIG (7 instances),	MIG (4 instances),	DLSS 3
	Transformer Engine, Sparsity 2:4	Sparsity 2:1	DLSS 4	

**Table 4:** Summary of Experimental Configuration.

Item	Specification
Host CPU	Intel Core i7-12700
Host OS	Windows
CUDA Version	12.4
Hashcat Version	v6.2.6
Attack Mode	Brute-force mask attack (-a 3)
Hash Type	WPA-PBKDF2-PMKID+EAPOL (-m 22000)
Charset Config.	Numeric only: ?d × *n*
	Num. + Upper: -1 ?d?u, ?1 × *n*
	Num. + All + Sym.: -1 ?d?l?u?s, ?1 × *n*
Runs per configuration	5
Max observed variance	≤6%
PSK length (*n*) tested	8, 9, 10 characters

**Table 5:** PSK Combination Search Space by Character Set.

Length	Numeric (N)	N + Letters	N + Letters + Symbols
8 digits	$10^{8}$	$2.82\times 10^{12}$	$6.10\times 10^{15}$
9 digits	$10^{9}$	$1.02\times 10^{14}$	$5.73\times 10^{17}$
10 digits	$10^{10}$	$3.66\times 10^{15}$	$5.39\times 10^{22}$

**Table 6:** Estimated Password Recovery Time on 4 × H100 GPUs (based on measured mean throughput; assumes uniform random password distribution).

PSK Length	Num	Num + Upper Letters	Num + All Letters + Symbols
8 digits	**11 s**	3 d 18 h	23 y 332 d
9 digits	**1 m 50 s**	130 d 13 h	2245 y 344 d
10 digits	18 m 46 s	12 y 316 d	—

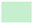
 Critically vulnerable, 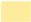 moderately resistant.

## Data Availability

The data presented in this study are available on request from the corresponding author.
